# Rapid and accurate recognition of erythrocytic stage parasites of *Plasmodium falciparum* via a deep learning-based YOLOv3 platform

**DOI:** 10.3389/fmicb.2025.1471436

**Published:** 2025-10-30

**Authors:** Wei He, Huiyin Zhu, Junjie Geng, Daiqian Zhu, Kai Wu, Li Xie, Jian Li, Hailin Yang

**Affiliations:** ^1^Key Laboratory of Industrial Biotechnology, Ministry of Education, School of Biotechnology, Jiangnan University, Wuxi, China; ^2^Department of Human Parasitology, School of Basic Medicine Science, Hubei University of Medicine, Shiyan, China; ^3^School of Internet of Things Engineering, Jiangnan University, Wuxi, China; ^4^Wuhan City Center for Disease Prevention and Control, Wuhan, China

**Keywords:** malaria, *Plasmodium falciparum*, you only look once, artificial intelligence, deep learning

## Abstract

**Background:**

Malaria remains a fatal global infectious disease, with the erythrocytic stage of *Plasmodium falciparum* being its main pathogenic phase. Early diagnosis is critical for effective treatment. This study developed and evaluated an artificial intelligence-assisted diagnosis (AI-assisted diagnostic) tool for malaria parasites.

**Methods:**

The peripheral blood samples of malaria patients were collected. Thin blood film smear were prepared, stained and examined by microscopic. After manual confirmation and validation with qPCR, the images of infected red blood cells (iRBCs) of *P. falciparum* were captured. Using a sliding window method, each original image was cropped into 20 small images (518 × 486 pixels). Selected iRBCs were classified, and *P. falciparum* was detected using the YOLOv3 deep learning-based object detection algorithm.

**Results:**

A total of 262 images were tested. The YOLOv3 model detected 358 *P. falciparum*-containing iRBCs, with a false negative rate of 1.68% (6 missed iRBCs) and false positive rate of 3.91% (14 misreported iRBCs), yielding an overall recognition accuracy of 94.41%.

**Conclusion:**

The developed AI-assisted diagnostic tool exhibits robust efficiency and accuracy in *Plasmodium falciparum* recognition in clinical thin blood smears. It provides a feasible technical support for malaria control in resource-limited settings.

## 1 Introduction

Malaria remains one of the top three public health diseases in the world, alongside AIDS and tuberculosis (Vassall and Masiye, 2022). There are five major *Plasmodium* parasites infected humans, including *Plasmodium falciparum*, *Plasmodium vivax*, *Plasmodium malariae*, *Plasmodium ovale*, and *Plasmodium knowlesi*. For the life cycle of *Plasmodium* spp. in human, it includes pre-erythrocytic stage and erythrocytic stage. For blood stage, it is the main pathogenic stage including rings, trophozoites, schizonts, and gametocytes. In 2021, there were an estimated 247 million cases of malaria worldwide, resulting in 619,000 deaths (WHO, 2022). Most deaths and severe cases are caused by *P. falciparum* malaria (Venkatesan, 2025). Unlike other *Plasmodium* species, *P. falciparum* exhibits rapid erythrocyte invasion and sequestration in microvasculature, which can lead to life-threatening systemic inflammation and vascular obstruction if not diagnosed promptly.

Early identification of malaria parasite species and the lifecycle of *Plasmodium* is imperative for precision treatment. As the gold standard for malaria diagnosis, microscopy is low-cost, high accuracy, and can identify the *Plasmodium* species and their life cycle (Hänscheid, 2003). However, hundreds of millions of blood smears are examined worldwide each year, which is a time-consuming and potentially error-prone process (Wilson, 2012). It requires a specially trained, experienced and skilled technician. Therefore, it is urgent to develop an intelligent recognition system that can automatically identify and classify malaria parasites to reduce work intensity and improve work efficiency.

Artificial intelligence (AI) is a computer technology that can be used to find the correlation of data information through techniques such as expressive learning, deep learning, and natural language processing, combined with computer algorithms, and can assist in clinical decision-making (He et al., 2019). At present, artificial intelligence has been successfully applied to CT image recognition of COVID-19 (Li et al., 2020), automatic analysis and diagnosis of microscope slide images (Smith et al., 2018), and association of genome sequence and proteomic profile with pathogen phenotype (Jamal et al., 2020; Lupolova et al., 2019).

In current study, it establishes a deep learning-based *Plasmodium* identification system to rapidly identify and classify malarial cell images, which will reduce the work intensity and improve efficiency for clinical treatment and malaria control.

## 2 Materials and methods

### 2.1 Sample collection and preparation

The blood of these *P. falciparum* patients was collected from migrant workers returning from African and Southeast Asian countries. These patients were first diagnosed by rapid diagnostic kits and qPCR, blood samples were made into thin blood smears, and microscope images were scanned and preserved. The details about preparation of blood smears are: Peripheral blood (2 μL) was collected from the patient to prepare thin smears (ensuring well-dispersed cells for morphological analysis). After air-drying, the smears were fixed with methanol and stained with Giemsa solution (pH 7.2) for 30 min, followed by rinsing with distilled water and drying. Imaging was performed using an Olympus CX31 microscope (100 × oil immersion objective, numerical aperture 1.30) equipped with a Hamamatsu ORCA-Flash4.0 camera. The image resolution was set to 2,592 × 1944 pixels with a uniform exposure time of 200 ms.

According to the acquired scanned malarial parasite cell images, and after the confirmation by the expert and qPCR results (Xie et al., 2020), the images of infected red blood cells (iRBCs) of *P. falciparum* were captured.

### 2.2 Data collection and preprocessing

The study protocol was approved by the Ethics Committee of the Wuhan Center for Disease Prevention and Control [Approval No.: (WHCDCIRB-K-2021013)].

#### 2.2.1 Image cropping and resizing

The original cell images obtained by scanning had a resolution of 2,592 × 1944 pixels, which is significantly larger than the 416 × 416 input size required by the YOLOv3 model. Direct input of unprocessed images would lead to loss of fine morphological features (e.g., Plasmodium nuclei and cytoplasm) critical for detection. Thus, a two-step preprocessing pipeline (cropping followed by resizing) was implemented:

A **Non-overlapping cropping**: A sliding window strategy was used to crop the original images into 518 × 486 sub-images. The window stride was calculated to ensure full coverage without overlap:
 o Horizontal stride = Original width ÷ 5 = 2,592 ÷ 5 = 518 pixels (matching the sub-image width), generating 5 horizontal sub-images per row.
 o Vertical stride = Original height ÷ 4 = 1944 ÷ 4 = 486 pixels (matching the sub-image height), generating 4 vertical sub-images per column.

This resulted in 20 non-overlapping sub-images (5 × 4 grid) per original image, avoiding redundant sampling while preserving complete spatial information.

B **Resizing and padding**: The 518 × 486 sub-images were resized to 416 × 416 to fit YOLOv3 input requirements, with strict preservation of aspect ratio to prevent morphological distortion:
 o First, the sub-images were proportionally scaled: the longer side (518 pixels) was resized to 416 pixels, and the shorter side (486 pixels) was scaled to 390 pixels, resulting in intermediate 416 × 390 images.
 o Black pixel padding (18 pixels on both top and bottom) was added to the 416 × 390 images to reach the 416 × 416 dimensions required for model input.

**Figure 1:**
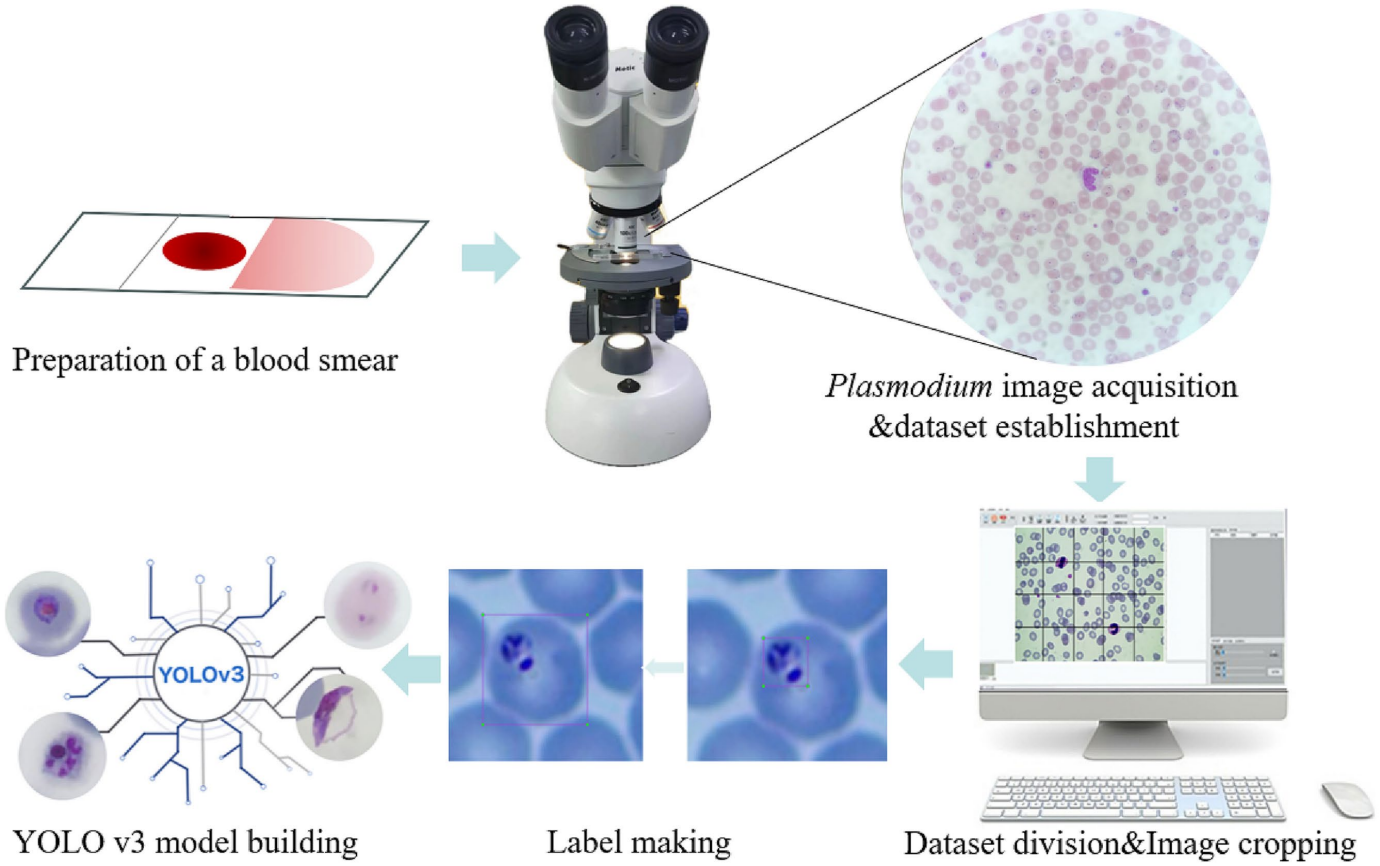
Schematic illustration of the recognition platform of *Plasmodium falciparum* via YOLO v3. The thin blood smears were scanned into *Plasmodium falciparum* images under a microscope, then the images were cropped and classified to establish a database, labels were created for each iRBC, and the YOLO v3 model was established.

The entire cropping process was implemented in Python, with three specialized libraries enabling automated and reproducible operation: Core cropping (Pillow), File traversal (os), Path management (pathlib.Path). The schematic diagram of the cropped image is shown in Figure 1.

#### 2.2.2 Label making

In object detection, the production of training labels directly affects the final detection results, so it is necessary to make labels for each cropped photo of malarial parasite cells, excluding images without malarial parasite cells to prevent them from affecting the final training results, and images that cannot be clearly determined as malarial parasites should be judged by professionals. In this work, considering that platelets and some impurities are highly similar in morphology and size to malarial parasite cells, to improve accuracy, single cells are taken as the detection object instead of recognizing single malarial parasites, so in the process of making labels, single cells containing malarial parasites need to be framed out. The schematic diagram of label making is shown in Figure 1.

#### 2.2.3 Dataset division

The dataset is divided into a training set, validation set and test set at a ratio of 8:1:1. The training set data are used to train the classification model, the validation set data are used to adjust the parameters of the model and optimize the model, and the test set data are used to test the classification performance of the model.

## 3 Model selection

### 3.1 General

Since the background color of the original image is inconsistent, we need to consider images with different background colors when selecting the test set to improve the reliability of the classification results.

To identify cells containing malarial parasites in the whole scanned image, the YOLOv3 algorithm is used in this work for recognition and processing. YOLOv3 is a one-step detection algorithm that directly inputs the picture into the network to extract the features of the whole picture and finally performs a regression operation on the whole picture to detect the target. The YOLOv3 algorithm directly divides the whole picture into nonoverlapping small blocks, avoiding a large number of sliding windows and improving the detection speed. YOLOv3 uses Darknet-53, which borrows the residual structure of ResNet to deepen the network structure while preventing the network from converging due to the gradient explosion. The use of residual blocks can prevent the loss of effective information and prevent gradient disappearance during the training of deep networks. In addition, there are no pooling layers in the network, which uses convolution with a stride of 2 to reduce the size of the feature map instead of pooling operations, which increases the accuracy of small object detection.

The core idea of YOLOv3 is multiscale prediction, borrowing the idea of pyramid feature maps, small feature maps for detecting large objects, and large feature maps for detecting small objects. YOLOv3 uses 3 scales, whose outputs are 52×52, 26×26 and 13×13 for detecting small, medium and large targets, respectively, with each scale predicting 3 anchor boxes. After adding the idea of multiscale prediction, YOLOv3’s ability to detect small targets has been enhanced. Specifically, when YOLOv3 processes the image, it divides the image into cells, and each cell predicts B bounding boxes and confidence scores. The confidence score consists of two parts: one is the possibility that the bounding box contains the target, denoted as Pr(object) , and when the bounding box contains the target Pr(object) =1, otherwise it is 0; the second is the accuracy of the bounding box, represented by the Intersection Over Union (IOU) $\mathrm{IO}U_{\text{pred}}^{\text{truth}}$ , so the confidence score can be expressed as $\mathrm{Pr}(\text{object})*\mathrm{IO}U_{\text{pred}}^{\text{truth}}$ . When classifying the target, each cell also predicts the probability values of the detected categories, i.e., the conditional probability under the condition of each bounding box confidence score, denoted as Pr(classi∣object) . The final prediction of YOLOv3 is a tensor of size Pr(classi∣object) , where S×S is the number of cells divided by the image, B is the number of bounding boxes in each cell, and C is the number of detected categories, which is much less than the number of sliding windows of two-stage detectors and is much faster in detection.

YOLOv3 avoids the gradient instability problem during training by directly predicting the width and height of the boundary box, which is done by applying a log space transformation or a simple offset to form the predefined default boundary box. Then, these transformations are applied to anchor boxes to obtain the predicted boundary boxes. YOLOv3 has three anchor boxes and can predict three boundary boxes for each cell. Where $b_{x},b_{y},b_{w},b_{h}$ are the center coordinates, width and height, $t_{x},t_{y},t_{w},t_{h}$ is the network output, $c_{x},c_{y}$ is the coordinates of the left top corner of the grid, and $p_{w},p_{h}$ are the dimensions of the anchor box. The YOLOv3 network uses the mean squared error as the loss function, which is composed of three parts: box localization error, IoU error of whether there is a target, and classification error. The loss function is shown in the following formula:


$$\begin{array}{l} \text{loss}=\lambda_{\text{coord}}\sum_{i=0}^{s^{2}}\sum_{j=0}^{B}i_{\mathrm{ij}}^{\mathrm{obj}}[{\left(x_{i}-{\hat{x}}_{i}\right)}^{2}+{\left(y_{i}-{\hat{y}}_{i}\right)}^{2}]\\ \;+\lambda_{\text{coord}}\sum_{i=0}^{s^{2}}\sum_{j=0}^{B}i_{\mathrm{ij}}^{\mathrm{obj}}[{\left(\sqrt{\omega_{i}}-\sqrt{{\hat{\omega }}_{i}}\right)}^{2}+{\left(\sqrt{h_{i}}-\sqrt{{\hat{h}}_{i}}\right)}^{2}]\\ \;+\sum_{i=0}^{s^{2}}\sum_{j=0}^{B}i_{\mathrm{ij}}^{\mathrm{obj}}{\left(c_{i}-{\hat{c}}_{i}\right)}^{2}+\lambda_{\text{coord}}\sum_{i=0}^{s^{2}}\sum_{j=0}^{B}1_{\mathrm{ij}}^{\mathrm{obj}}{\left(c_{i}-{\hat{c}}_{i}\right)}^{2}\\ \;+\sum_{i=0}^{s^{2}}l_{\mathrm{ij}}^{\mathrm{obj}}\sum_{c\in \text{classes}}{\left[p_{i}(c)-{\hat{p}}_{i}(c)\right]}^{2} \end{array}$$

where the first and second terms indicate the weight of the prediction box localization error and the center coordinate error, respectively, S represents the number of grids divided into the image, B represents the number of predicted boxes for each grid, $i_{\mathrm{ij}}^{\mathrm{obj}}$ represents whether the j predicted box of grid i detects the target, and x,y,ω,h represent the center coordinates and width and height of the true box, respectively. The third and fourth terms indicate the IoU error, c represents the confidence score, the last term indicates the classification error, and $p_{i}(c)$ represents the conditional probability that the detected target belongs to C .

When using object detection for model prediction, a large number of overlapping predicted boxes will appear. Nonmaximum suppression (NMS) can be used to deduplicate the large number of overlapping predicted boxes output by the object detection model. NMS first selects the detection box with the highest confidence as the best prediction boundary for the target coordinates, then deletes it from the detection box list and adds it to the final detection box list. Two detection boxes with an overlap degree greater than the threshold are often duplicate inspections of the same target object and should be removed, while detection boxes with an overlap degree less than the threshold indicate a correct detection of the target and should be added to the final detection box list.

### 3.2 Training the model

Once the training model is set up, use the training set images to train the model. In the training stage, it is necessary to set a suitable batch size, set the learning rate and learning rate adjustment strategy, set the optimization algorithm, and use a suitable parameter initialization method and training rounds. In the process of model training of YOLOv3, the batch size is set to 16, It is a *power-of-two* value selected to fit within the 16 GB video RAM (VRAM) of the training GPU while optimizing parallel computation efficiency. The learning rate was initialized with a maximum of 10^−2^ and decayed to a minimum of 10^−4^ using a cosine descent schedule; this scheduler was adopted to facilitate stable convergence without extensive manual hyperparameter tuning. Additionally, the model was trained for up to 300 epochs, with early stopping triggered if no significant improvement in validation accuracy was observed for 50 consecutive epochs.

### 3.3 Validating the effectiveness of the model

After each training epoch, the model was validated using the validation set to monitor its generalization capability. When validation performance plateaued or declined, hyperparameters (e.g., learning rate, batch size) were adjusted based on empirical observations:

- The learning rate was optimized via a cosine descent schedule, with initial trials testing configurations (0.01–0.001, 0.001–0.0001) to balance convergence speed and stability.
- The batch size of 16 was retained to fit within the GPU memory constraints (16 GB VRAM), as larger powers-of-two values caused memory overflow.

This iterative tuning process continued until the model achieved peak classification accuracy on the validation set, defined as no significant improvement over 50 consecutive epochs (early stopping criterion). The optimal model weights were then saved, concluding the training stage after a maximum of 300 epochs.

### 3.4 Saving the best model

The best classification model obtained through training is saved, and the model and model weight files are saved so that the weights can be loaded into the model when predicting, and the classification results can be obtained by inputting the predicted image. The overall technical roadmap is shown in Supplementary Figure S1.

## 4 Results

### 4.1 Sample preparation and dataset construction

Totally, 371 blood samples were collected from June 2011 to December 2019. The 307 molecularly identified *P. falciparum* samples were used for this study. A total of 1,252 captured images containing infected red blood cells were obtained from microscope. From these samples, 929 specimens were selected for this study. All of which had been molecularly identified as *Plasmodium falciparum* and accompanied by scanned microscopic images.

To establish a usable image database for model training and testing, the 929 scanned microscopic images of *P. falciparum* were subjected to an image cropping process. After cropping, all images in the database were manually labeled to support the subsequent *P. falciparum* recognition tasks (e.g., labeling infected red blood cells [iRBCs] and distinguishing impurities).

### 4.2 Evaluation of YOLOv3 model adaptability

**Figure 2:**
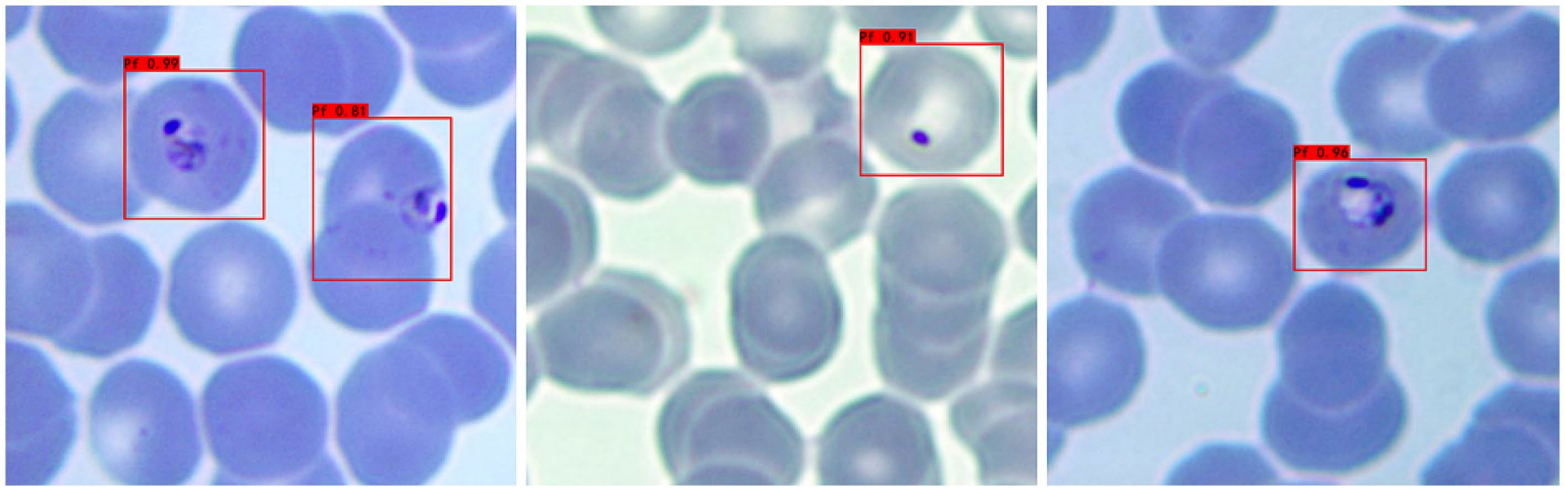
Recognition results of *Plasmodium falciparum* parasites under different backgrounds by YOLOv3.

**Figure 3:**
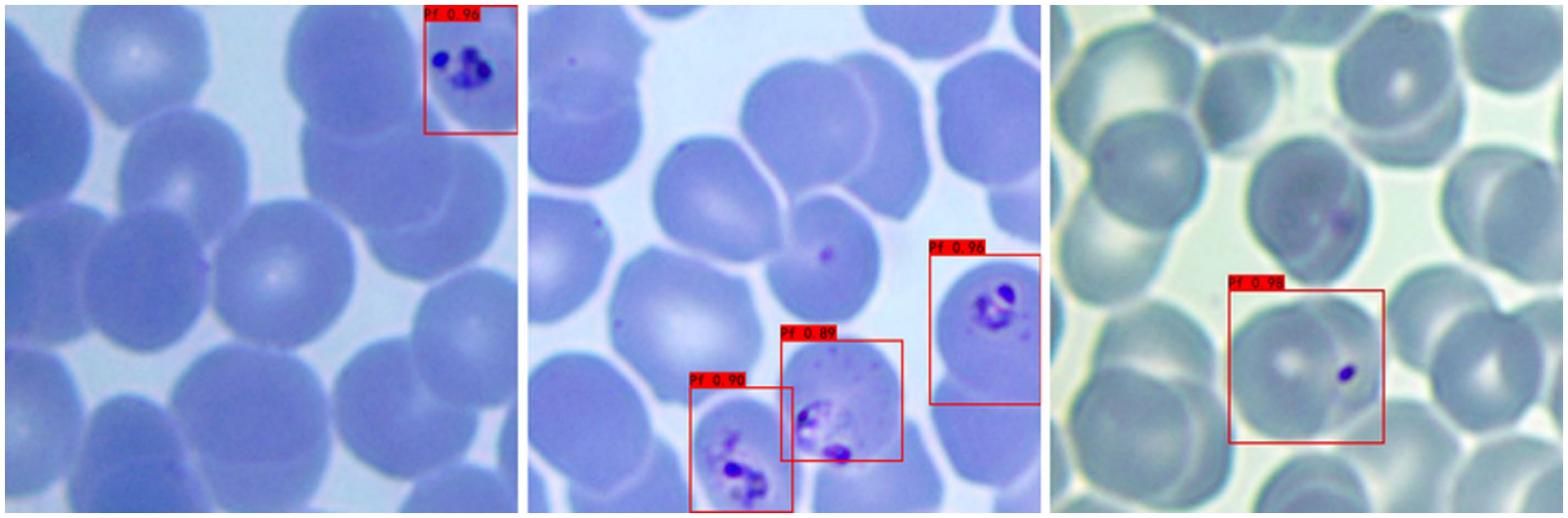
Recognition results of *Plasmodium falciparum* parasites of different erythrocytic shapes by YOLOv3.

**Figure 4:**
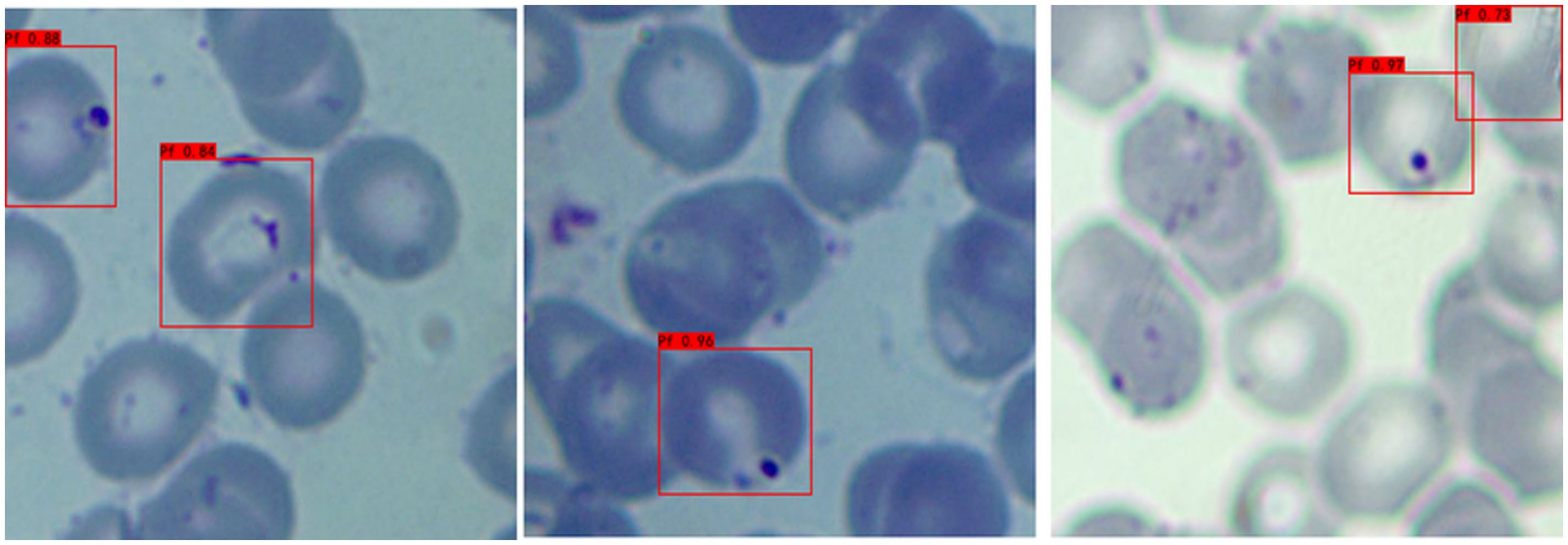
Recognition results of *Plasmodium falciparum* parasites with impurities by YOLOv3.

**Figure 5:**
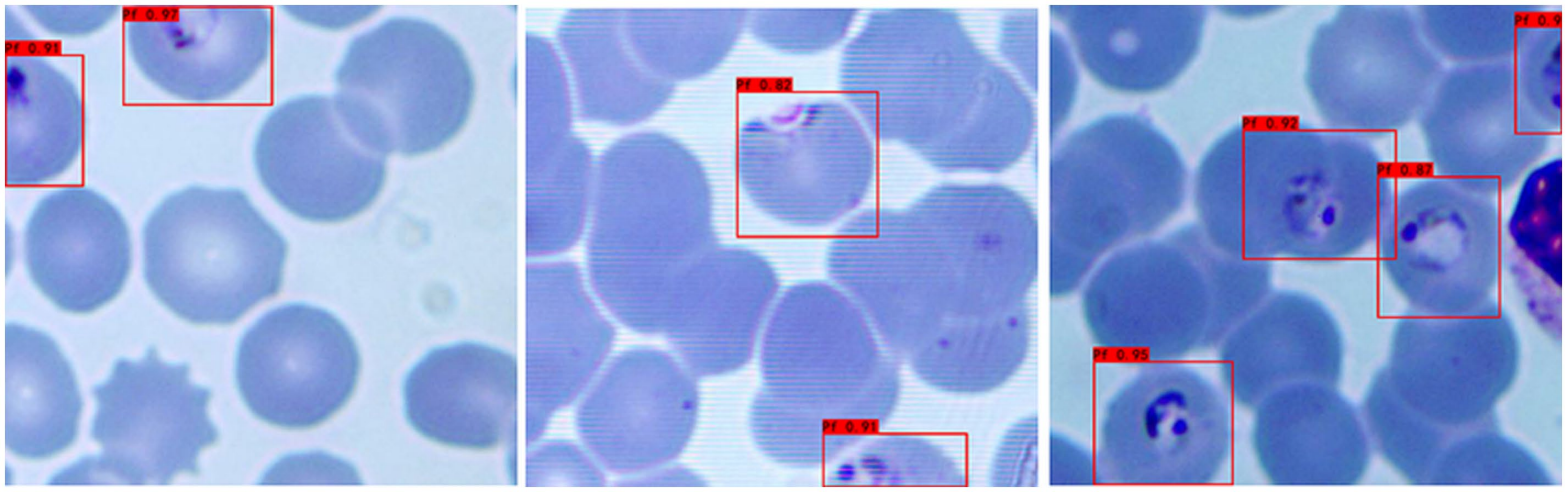
Recognition results of *Plasmodium falciparum* parasites incomplete cell recognition results by YOLOv3.

a **Adaptability to different background colors:** In this experiment, to increase the credibility of the results, we selected iRBCs with different background colors for testing, and the test results are shown in Figure 2. Under different background colors, the YOLOv3 network can obtain the same accurate results.
b **Adaptability to different *P. falciparum* morphologies:** Since iRBCs have different morphologies in different growth cycles, it is necessary to test *Plasmodium* of different morphologies, and the same satisfactory results can be obtained; the recognition results are shown in Figure 3.
c **Distinguishing ability for impurities:** In the process of recognizing iRBCs, due to their morphology and color being similar to *Plasmodium*, impurities such as platelets will have a great influence on the experimental results. Therefore, whether these impurities can be successfully distinguished from *Plasmodium* is an important criterion for judging the quality of recognition results. Figure 4 shows the recognition results of *Plasmodium* images with impurities. YOLOv3 successfully distinguishes impurities without misjudgment.
d **Recognition of incomplete cells:** In the process of image cropping, it is necessary to divide an image into two parts. YOLOv3 also successfully recognizes these incomplete cells, avoiding missed inspection, as shown in Figure 5.

### 4.3 Initial testing performance of the YOLOv3 model

In this experiment, a total of 297 images were tested, and the YOLOv3 model detected a total of 366 cells containing *Plasmodium*, of which 9 iRBCs were missed, with a missed rate of 2.46%, 38 cells were misreported, with a misreported rate of 10.38%, and the overall recognition accuracy was 87.26%. The main reason for the low accuracy is inaccurate labeling, which confuses some impurities with *Plasmodium*, resulting in a large number of cells containing impurities being misjudged as cells containing *Plasmodium* in the test process.

### 4.4 Model optimization and improved testing performance

To address the labeling-induced accuracy issue, we optimized the workflow by having professionals proofread all manual labels. After proofreading, we further expanded the dataset: from 2,792 returned cropped images (derived from the original 929 scanned images), 179 images without any valid labels were excluded, resulting in a final labeled dataset of 2,613 images. This dataset was randomly divided into a training set, validation set, and test set at an 8:1:1 ratio for retraining the YOLOv3 model.

A second test was conducted using 262 labeled cropped images (from the optimized test set), with the following improved results:

- Total *P. falciparum*-containing cells detected by the optimized YOLOv3 model: 358
- Missed detection: 6 cells (missed rate = 1.68%)
- False positive cells: 14 (misreported rate = 3.91%)
- Overall recognition accuracy: 94.41%

**Figure 6:**
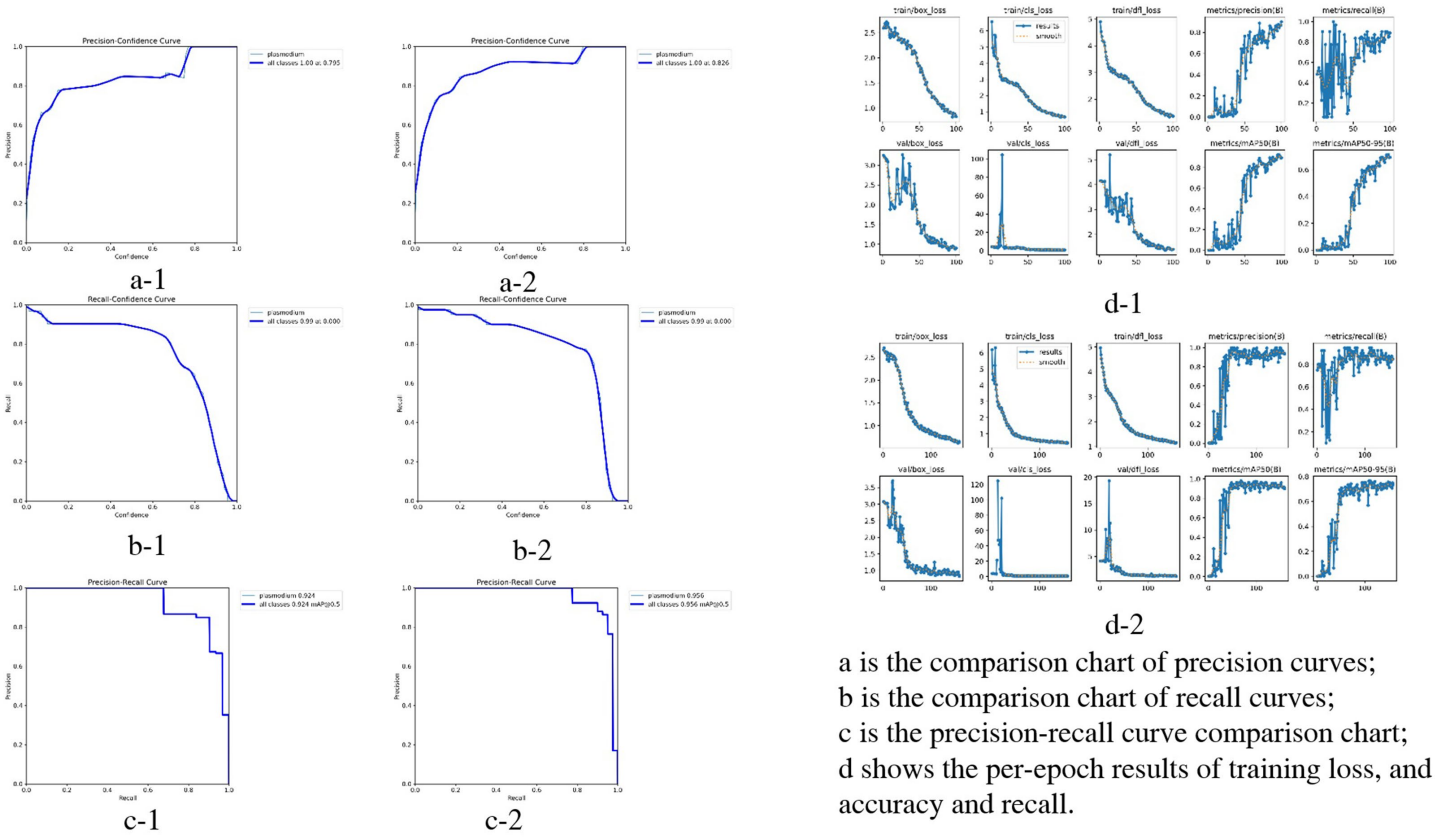
Performance comparison of YOLOv3 model in *Plasmodium falciparum* detection: first vs. second experiment.

Compared with the initial test, the optimized model showed significant improvements in all performance metrics. Detailed comparisons between the two test groups (initial test: a-1, b-1, c-1, d-1; optimized test: a-2, b-2, c-2, d-2) are presented in Figure 6.

**Figure 7:**
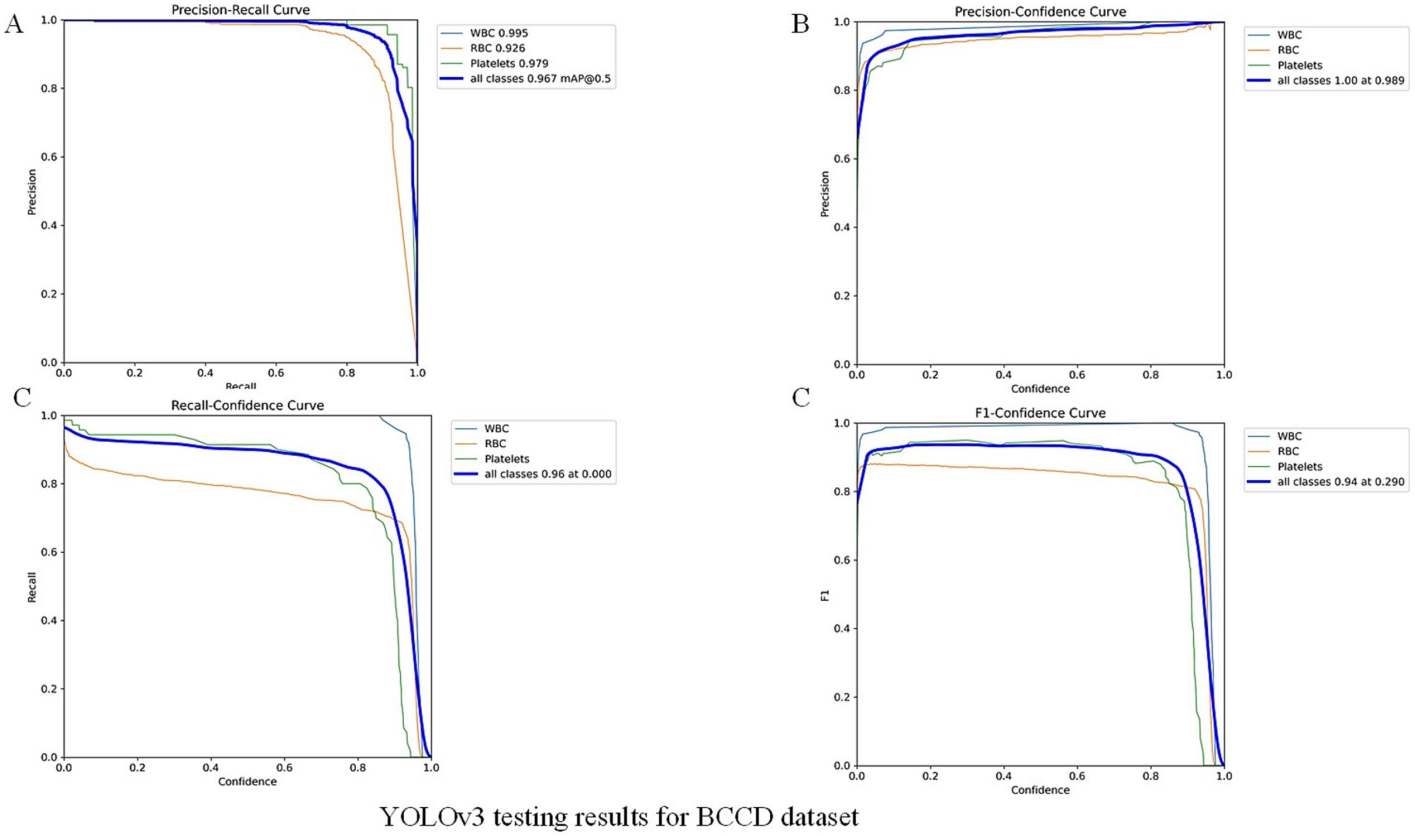
YOLOv3 testing results for BCCD dataset.

To further validate our module, we evaluated the detector on the public BCCD (Blood Cell Count and Detection) dataset with light fine-tuning. Fine-tuning followed the YOLOv3 training protocol used in this study: up to 300 epochs with early stopping if no improvement was observed for 50 consecutive epochs. On BCCD the detector achieved mAP = 96.7%, F1 = 94%, Recall = 96% and Precision = 100% (full metrics and training details are provided in the Figure 7 -YOLOv3 testing results for BCCD datasets). These supplementary results contrast with the overall accuracy reported above for our clinical thin-smear dataset (94.41%). Notably, our 94.41% overall accuracy is slightly lower than the 96% + metrics reported in studies such as Abdurahman et al. (2021) and Chibuta and Acar (2020). This difference is not due to model limitations but to fundamental disparities in dataset complexity. While we do not claim universal state-of-the-art performance. Reported metrics in other YOLO-based malaria studies (e.g., Abdurahman et al., 2021; Yang et al., 2020) were obtained on different datasets (often thick smears or controlled images) and are therefore not directly comparable.

## 5 Discussion

### 5.1 Limitations of existing malaria diagnostic methods

Currently, malaria diagnosis mainly relies on microscopic examination, rapid diagnostic tests (RDTs) and PCR-based molecular examination. RDTs are serological antibody detection methods based on enzyme-linked immunosorbent assays (ELISAs). RDTs provide a qualitative diagnosis by detecting one or more *Plasmodium* proteins, such as histidine-rich protein-2 (HRP-2), lactate dehydrogenase (LDH), aldolase, *etc* (Cunningham et al., 2019). RDTs do not require highly trained staff and laboratory support, and they are currently the most commonly used immunological assay (Odaga, et al., 2014). Meanwhile, low density and the inability of parasites to produce HRP2 can lead to false negative RDT results (Cunningham et al., 2019), and RDTs are deficient in identifying Plasmodium species. Compared with RDTs and microscopy, molecular biological assays have excellent analytical sensitivity and specificity (Berzosa et al., 2018; Cunningham et al., 2019; Golassa et al., 2013)and can identify antimalarial drug resistance (Apinjoh et al., 2019). However, due to its high cost, complex operation, difficult personnel training and special instruments, it is difficult to popularize its practical application in grassroots units.

The combination of microscopy and artificial intelligence will be promising in the field of malaria diagnosis. However, due to the diversity and polymorphism of *Plasmodium* morphology and the difference in blood smear operation, it has been difficult in the field of medical identification and detection. For YOLO, it is a convolutional neural network (CNN) that takes an input image and learns category probabilities and bounding box coordinates (Odaga, et al., 2014). It is designed for fast and accurate object detection and is suitable for real-time use. It uses a single convolutional neural network to predict object classes and find their locations (Odaga, et al., 2014).

### 5.2 Innovation and validation of the model in this study

In this study, we applied a YOLOv3-based deep learning model to detect *P. falciparum* in red blood cells (RBCs). A two-step classification approach yielded recognition accuracies of 87.26 and 94.41% after expert refinement, underscoring the potential of our framework as a rapid and robust alternative to traditional microscopy. While Abdurahman et al. (2021) reported 96.32% mAP for malaria parasite detection using modified YOLO architectures, direct performance comparison with our study is methodologically inappropriate due to fundamental differences in detection targets and dataset characteristics. Their study focused on thick blood smears, where RBCs are lysed, creating relatively simple backgrounds with primarily parasites and WBCs. Our study addresses the more clinically essential but computationally challenging task of detecting *P. falciparum* in thin blood smears with intact RBCs. Thin blood smear analysis presents significantly greater challenges: (1) complex backgrounds with numerous overlapping intact red blood cells; (2) lower parasite density per field; (3) multiple interference factors including platelet aggregation, staining artifacts, cell debris, and precipitates commonly encountered in clinical practice; and (4) greater morphological diversity. These factors make thin smear parasite detection fundamentally different and more complex computer vision task than thick smear detection. Furthermore, Model performance metrics are inherently task-specific and dataset-dependent; therefore, the apparent 1.91% difference reflects different detection challenges rather than comparative model effectiveness.

The supplementary experiment in public dataset indicates that the high performance on BCCD primarily reflects the detector’s strong capability under standardized, low-variance imaging conditions (BCCD: mAP = 96.7%). BCCD is a controlled blood-cell dataset with relatively homogeneous backgrounds and stable imaging settings. By contrast, our clinical thin-smear images contain substantial real-world interferences (e.g., leukocytes, platelets, staining artifacts, and variable microscope settings), which substantially increase detection difficulty and result in lower measured performance (clinical thin smear: overall accuracy = 94.41%). Thus, the principal source of performance differences is dataset complexity and task variation: controlled datasets demonstrate capability in idealized conditions, while clinical thin smears reflect real-world challenges. Additionally, public datasets based on thick smears [e.g., those used by Abdurahman et al. (2021)] represent a different detection task and should not be directly compared numerically with thin-smear results. In summary, the BCCD experiment demonstrates detector performance under standardized conditions, whereas our clinical evaluation highlights applicability and robustness in realistic, challenging settings. Systematic cross-domain transfer and domain adaptation studies are planned as future work.

### 5.3 Comparative analysis with other studies

Compared with other thin smear detection studies, Yang et al. (2020) achieved 93.46% accuracy for *malaria parasite* detection, while Hung et al. (2020) reported 98% accuracy using computationally expensive cascaded Faster R-CNN with AlexNet for *P. vivax*, where parasitic objects are larger than *P. falciparum*. Muhammad et al. (2025) reported mAP 80.3% based on YOLOv8 with public datasets and Lufyagila et al. (2025) get mAP 86.2% based on public clinical images. Our 94.41% accuracy for *P. falciparum* in thin clinical smears represents robust performance for this challenging task while maintaining real-time inference speeds suitable for resource-constrained clinical environments. Meanwhile, we have conducted an extensive review of recent deep learning-based malaria detection research, particularly those employing YOLO and related techniques. The key findings are summarized in Table 1.

**Table 1:** Comparison of recent YOLO-based and related deep learning malaria detection studies.

Study (Year)	YOLO version/Model type	Detection object (Smear type)	Dataset size and source	Key performance metrics	Highlights and limitations
Chibuta and Acar (2020)	Modified YOLOv3	Thick smear	Clinical/size N/A	Accuracy: 96.5%	Improved small-object detection; strong performance on thick smears; not adapted for thin smear morphology.
Abdurahman et al. (2021)	YOLOv3 / YOLOv4-MOD	Thick smear	Public (1780–2,300 imgs)	YOLOv4-MOD mAP@0.3: 96.32%	Engineering-oriented design emphasizing CPU/GPU/NCS2 deployment; lacks complex thin-smear testing.
Paul et al. (2022)	YOLOv5	Thin smear	Public (1,182 imgs)	mAP@50: 79%	Enhanced small-object and cross-species generalization; mostly public datasets, not clinical-grade images.
Zedda Lucas et al. (2024)	YOLO-SPAM/PAM	Thin smear and cropped cells	Public datasets	mAP@50 varies 67.4–96%	Dual detection for clinical parasitemia estimation; remains thick-smear–based.
Özbilge et al. (2024)	YOLOv8	Thin smear	Private (1,081 imgs)	mAP@50: 90.3%	First to address thin-smear rouleaux morphology; performance limited by complex cell stacking and artifacts.
Muhammad et al. (2025)	YOLOv9	Thin smear	Private	mAP@50: 80.3%, Recall: 76.6%	Emphasizes multi-species, multi-stage end-to-end detection; public dataset–based generalization.
Lufyagila et al. (2025)	YOLOv11m	Thick smear	Public clinical (2,310 imgs)	mAP@50: 86.2%	Real-world African thick smears; parasite & WBC co-detection; less challenging visual background.

We can clearly delineate the key differences and innovations of this study compared to existing works:

1 **Focus on Object Detection in the Most Challenging Real-World Clinical Thin Blood Smear Scenarios**: We clearly differentiate our approach from studies primarily focused on thick smears (Abdurahman et al., 2021; Chibuta and Acar, 2020; Lufyagila et al., 2025) or those solely on classification (e.g., Chibuta and Acar, 2020). We highlight the significantly greater computational challenges of precise object detection in thin smears due to complex backgrounds, lower parasite density, and prevalent clinical interference factors, especially in our large-scale clinical dataset.
2 **Emphasis on Data-Driven Optimization Rather than Solely Model Architecture Iteration**: Unlike many studies that pursue the latest YOLO versions (Lufyagila et al., 2025; Muhammad et al., 2025; Özbilge et al., 2024) or incorporate complex architectural modifications (Abdurahman et al., 2021), this study intentionally opted for the classic YOLOv3 architecture. Our core innovation and improvement strategy lies in a deep understanding and optimization of data quality. We invested significant effort in addressing pervasive “labeling issues” within clinical datasets (including inconsistencies, missed labels, and incorrect labels) and employed adaptive data augmentation and training strategies to enhance model performance in complex real-world scenarios. This strategy demonstrates that, for specific clinical applications, a deep understanding and meticulous optimization of dataset quality, coupled with training strategies adapted to data characteristics, can contribute as much, if not more, to model performance and practicality than mere architectural iteration. This “data-first” optimization pathway provides significant guidance for clinical deployment in resource-limited settings or where high model stability and reliability are paramount, and effectively mitigates potential limitations of relatively “older” model architectures when facing complex real-world data.
3 **Demonstration of Excellent Generalization Ability and Clinical Relevance through Cross-Dataset Validation:** To further validate the model’s robustness and generalization ability, we applied the model trained on our clinical thin blood smear dataset, without any additional fine-tuning, to the general BCCD dataset. On this dataset, our YOLOv3 model achieved excellent performance with mAP of 96.7%, Precision of 100%, Recall of 96%, and F1 of 94%. This result holds dual significance:

a **Strong Generalization Capability:** It powerfully demonstrates that a model trained on extremely complex and interference-rich clinical thin blood smear data possesses remarkable cross-dataset generalization ability. The model not only learned to identify malaria parasites but also acquired core visual features for precisely localizing and recognizing tiny objects in noisy backgrounds, allowing it to adapt efficiently to other blood cell images with simpler structures.
b **Highlighting the Challenge of Clinical Data:** The high performance on the BCCD dataset retrospectively confirms the high challenge level of our primary clinical dataset. Achieving such high scores on a relatively clean dataset like BCCD, with distinct object features, further underscores the difficulty and practical significance of obtaining a 94.41% mAP on clinical data laden with real-world interferences.

Our methodological contributions include: (1) validation on real clinical data with authentic interference factors; (2) two-stage classification with expert refinement demonstrating practical human-in-the-loop validation; (3) incomplete cell recognition capability crucial for practical deployment; (4) computational efficiency prioritizing clinical applicability and (5) build the fundamental for stage-specific classification of *P. falciparum*. These features position our system for effective deployment in endemic regions where thin smear examination remains the gold standard for species identification and parasitemia quantification. We have therefore refrained from claiming state-of-the-art performance. Differences in smear type (thin vs. thick), dataset control (clinical vs. curated/controlled), image acquisition (microscope settings, smartphone vs. slide scanner), and evaluation metrics can substantially affect reported numbers; comparisons must account for these factors. We cite representative YOLO-based malaria detection studies (Abdurahman et al., 2021; Yang et al., 2020) and discuss their dataset/task differences in the previous work section.

### 5.4 Performance comparison with new generation YOLO models

**Figure 8:**
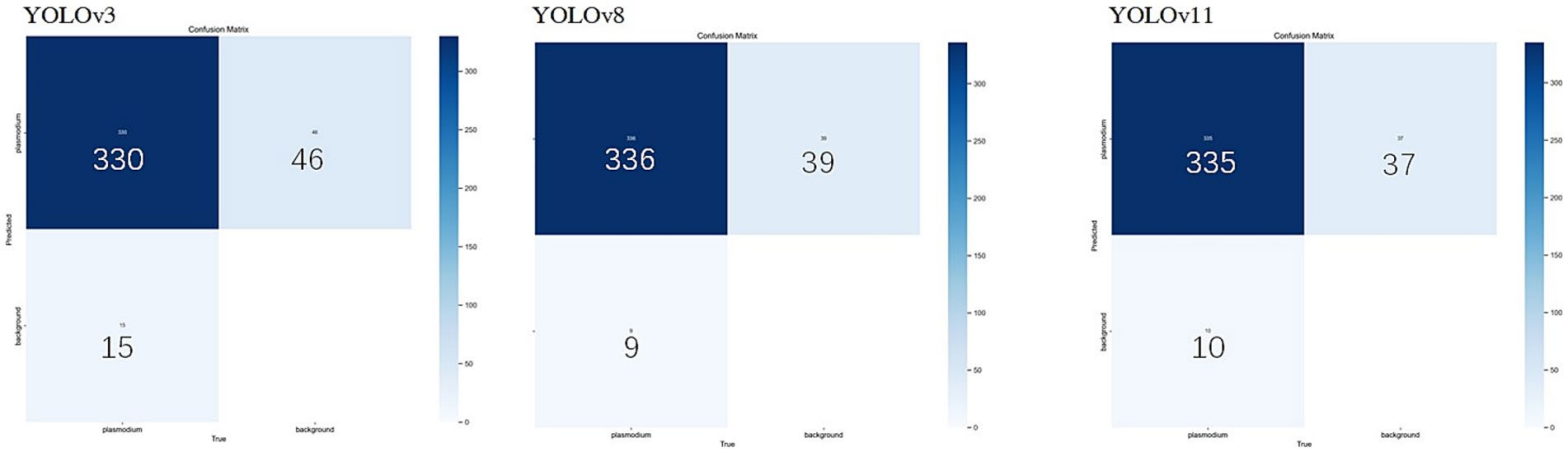
YOLOv3, YOLOv8, YOLOv11 mixed model confusion matrix.

**Figure 9:**
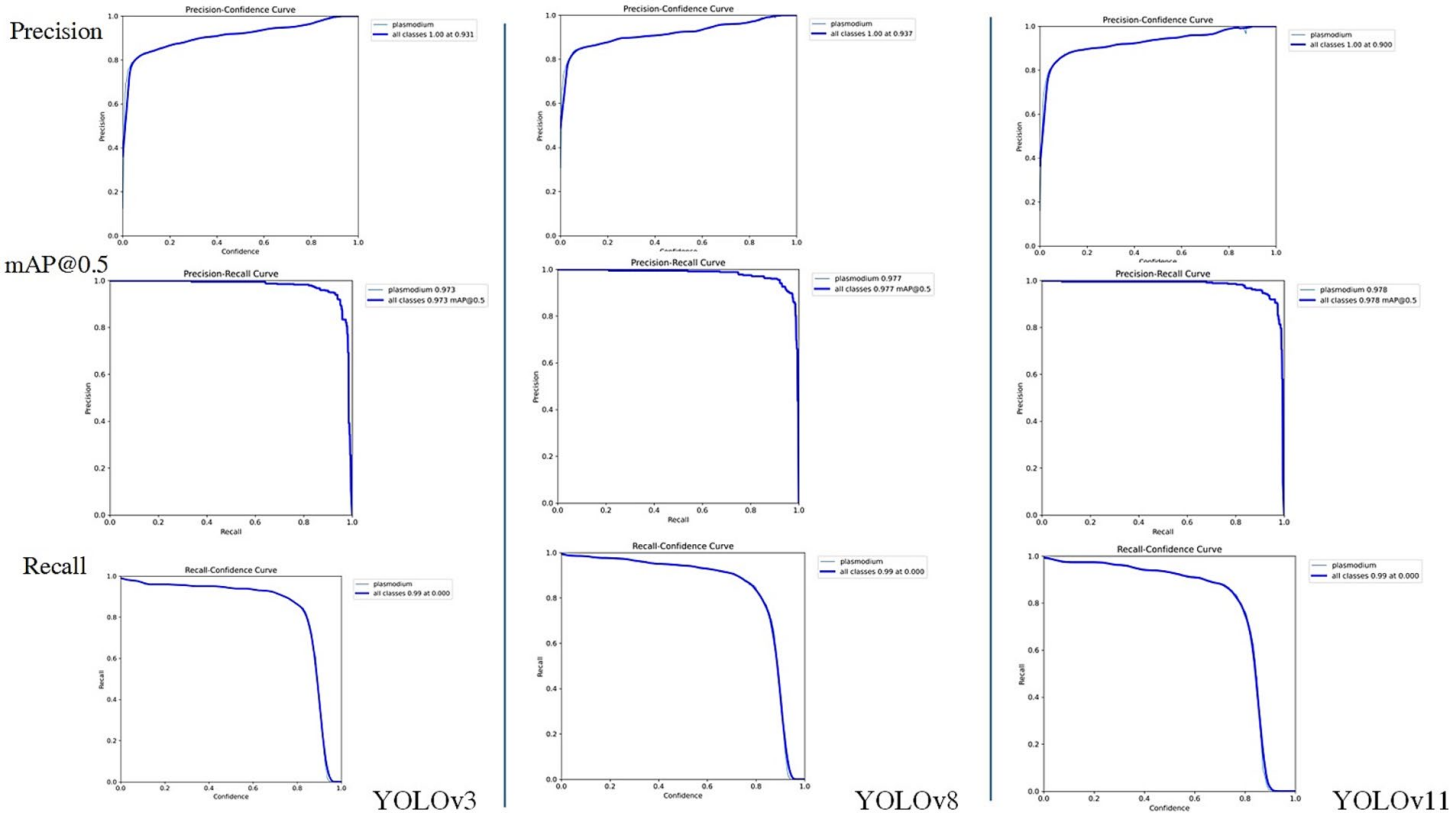
The Accuracy, mAP@50 and recall comparation among YOLOv3, YOLOv8 and YOLOv11.

Considering the YOLO series of object detection algorithms have undergone several significant iterations and performance improvements. To further verify the continued superiority of the YOLO series in the field of microscopic image recognition and to investigate its technological development trends, we conducted additional comparative experiments using the same dataset as in this study’s YOLOv3 experiments (selected from 2,613 optimized training images), based on the YOLOv8 and YOLOv11 models. The test results are shown in Figures 8, 9. The comparation results in showed in Table 2.

**Table 2:** Performance comparison of YOLOv3, YOLOv8 and YOLOv11 models.

Module	Recall	Precision	mAP@0.5	Testing image numbers	TP (True positives)	FP (False positives)	FN (False negatives)
YOLO v3	0.943	0.943	0.973	262	290	6	14
YOLO v8	0.918	0.955	0.977	262	290	48	0
YOLO v11	0.912	0.962	0.978	262	290	41	0

Through comparison, on the same clinical thin blood smear dataset, YOLOv8 and YOLOv11 achieved mAP@0.5 scores of 0.977 and 0.978 respectively, showing slight improvements over YOLOv3’s 0.973. The enhancement in precision metrics (2–3%) was particularly significant, reflecting the new models’ advantages in reducing false detections. However, the recall rates of the new models (0.918 and 0.912) were lower than YOLOv3 (0.943), indicating that while maintaining high precision, they may sacrifice the detection rate of some parasites. Confusion matrix analysis revealed that YOLOv8 and YOLOv11 had zero missed detections, but their false detection counts were significantly higher than YOLOv3, suggesting increased sensitivity to non-parasitic structures in complex backgrounds. Notably, compared to YOLOv3, the new generation models reduced parameter count and computational load by nearly 90%, significantly improving inference speed and deployment flexibility while maintaining accuracy. This balance between performance and efficiency provides crucial technical support for promoting microscopic image detection in resource-constrained medical environments.

### 5.5 Clinical application prospects and future direction

Although the present work focused on binary classification of infected versus uninfected erythrocytes, it did not attempt to differentiate among the four intraerythrocytic stages of *P. falciparum* (ring, trophozoite, schizont, and gametocyte). However, stage-level classification carries important clinical and epidemiological implications. Microscopy-based staging remains the cornerstone for estimating parasite density and disease severity, as higher proportions of trophozoites and schizonts are associated with severe malaria, while gametocyte detection is critical for assessing transmission potential (Ashley et al., 2018; White, 2018). Moreover, the efficacy of many antimalarial drugs is stage-dependent, making precise staging valuable for therapeutic monitoring (Ashley et al., 2018).

Recent advances in AI demonstrate the feasibility of stage-specific classification. Convolutional neural networks (CNNs) have achieved high accuracy in distinguishing between erythrocytic stages using thin smear images (Muhammad et al., 2025), while mobile-based AI platforms have also shown promise in field-deployable stage recognition (Yang et al., 2020). Incorporating stage-specific annotations into the training dataset, together with sufficient sample diversity, will be essential to enable robust multiclass recognition across varying smear preparations. Such developments could enhance malaria case management, provide more precise monitoring of drug response, facilitate surveillance of transmission-blocking interventions, and support malaria elimination programs.

In addition to enhancing conventional microscopy, the proposed YOLOv3-based system holds promise as a complementary component of broader diagnostic strategies. Its ability to detect low-parasite-density infections in digitized smear images makes it well suited for integration with other tools such as rapid diagnostic tests (RDT) or molecular assays (PCR). In low transmission settings, AI-assisted microscopy could be deployed for initial screening, followed by confirmatory testing using alternative methods. This combined approach may improve diagnostic sensitivity, reduce the dependence on highly experienced microscopists, and ultimately aid malaria control efforts in resource-limited environments.

Meanwhile, the present findings should be considered preliminary and are limited to data collected from a single clinical center. Although the dataset underwent rigorous expert annotation and quality control, the generalizability of the model to diverse patient populations, imaging conditions, and microscope hardware remains to be validated. Future work will focus on multi-center studies involving larger and more heterogeneous datasets to assess robustness across different clinical environments. Comprehensive validation in such settings will be essential before considering deployment of the model in routine clinical workflows.

In conclusion, our YOLOv3-based framework demonstrates the feasibility of applying real-time object detection for malaria diagnosis and offers a powerful complement to classical microscopy. Future work should aim to expand the dataset to include multiple *Plasmodium* species, integrate stage-level classification, and validate the system across diverse clinical settings. Continued development of AI-powered malaria diagnostic platforms will rely on high-quality annotated datasets, algorithmic innovation (such as Yolo V5, V8, etc.), and rigorous clinical validation to maximize their translational potential in both endemic and resource-limited environments.

## 6 Conclusion

The developed AI diagnostic tool based on YOLOv3 has high recognition efficiency and accuracy for the identification and classification of *P. falciparum* malaria parasites. The overall recognition accuracy reached 94.41%. This tool provides a feasible technical support for malaria control in resource-limited setting, with performance competitive for clinical thin smear detection but not claimed as state-of-the-art across all malaria diagnostic scenarios.

## Data Availability

The original contributions presented in the study are included in the article/Supplementary material, further inquiries can be directed to the corresponding author/s.
