# Supplementary figures and images for: Rapid and accurate recognition of erythrocytic stage parasites of Plasmodium falciparum via a deep learning-based YOLOv3 platform
Source: Front Microbiol. 2025 Oct 30;16:1471436. doi: 10.3389/fmicb.2025.1471436 (PMC12611930; doi:10.3389/fmicb.2025.1471436)

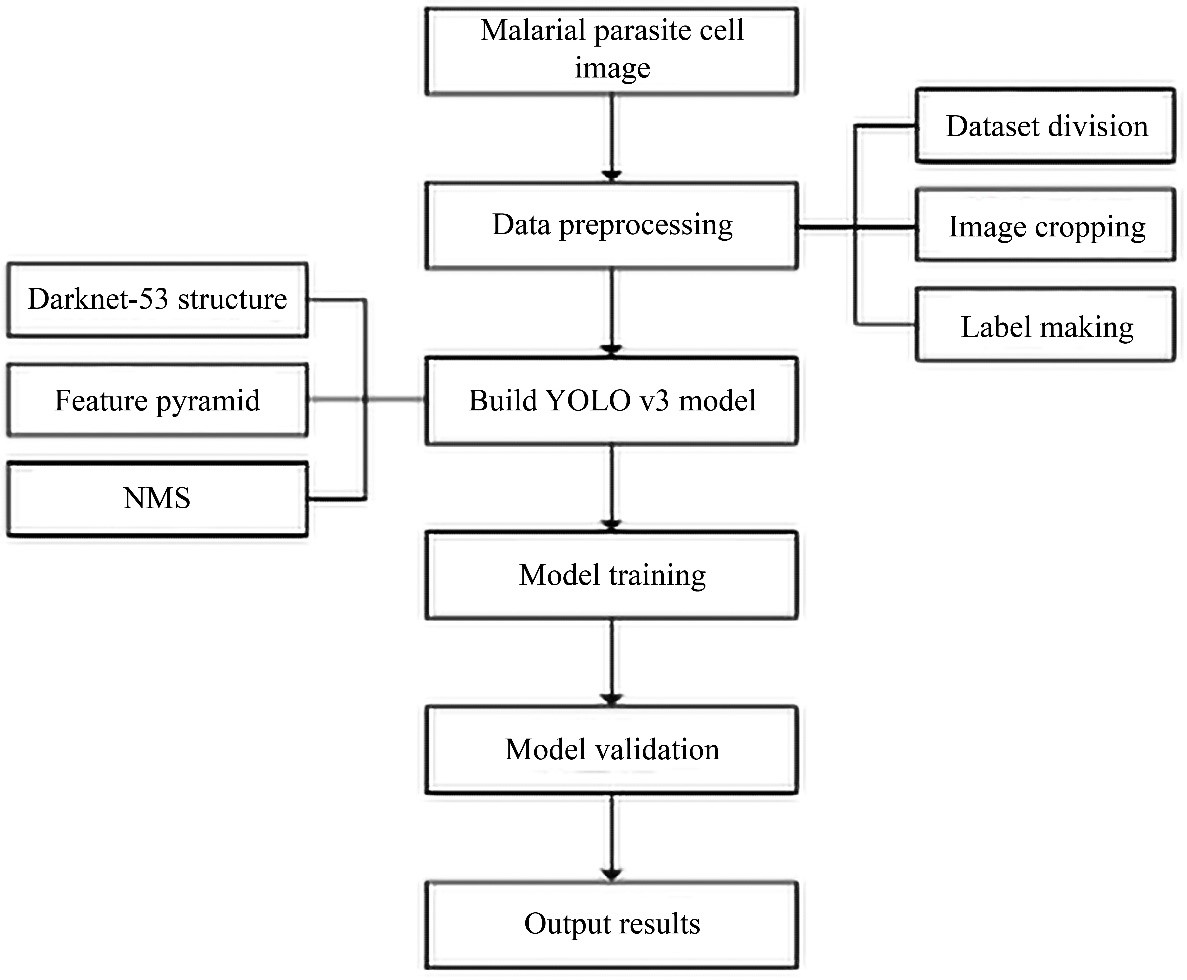

Supplement: Supplementary file 1 [file Image_1.JPEG]
